# Hernia of the Cornea—Action of Eserine and Pilocarpine

**Published:** 1878-12

**Authors:** F. C. Hotz


### Original Lectures.

### HERNIA OF THE CORNEA—ACTION OF ESERINE AND PILOCARPINE.

(*Clinical Lecture delivered at the Illinois Charitable Eye and Ear Infirmary, October* 9, 1878.)

By F. C. Hotz, M. D.

Gentlemen: The right eye of this boy exhibits a peculiar affection of the cornea, which, though not very common, is practically so important that it may well occupy your attention for a few moments. Without at present taking any notice of the granular condition of the conjunctiva, and the faint pericorneal redness, you will direct your attention to the cornea. This membrane has lost its brilliant lustre; its surface is dull, uneven, hazy, and traversed by fine, slightly tortuous blood vessels. In the lower half of the cornea the cloudiness is like a uniform thin mist through which you can still pretty well distinguish the color of the iris and the lower margin of the pupil. In the upper half the cloudiness increases in density to form a grayish white opacity. And in the center of this opacity, about midway between the center and upper margin of the cornea, you observe a small, oval, shining spot of the size of a pin's head. When you look at it from the front, it appears jet black, but when you inspect it from the side, you notice that it is a perfectly transparent vesicle which projects a little above the anterior surface of the cornea. You will further notice that through this small vesicle, the color and design of the iris appears better defined than through the lower half of the cornea; indeed, you can see it almost as distinctly as through a normally transparent cornea.

Focal illumination shows better than daylight the perfect transparency of this minute portion of the cornea.

This peculiar phenomenon of a transparent and projecting spot in the midst of a dense opacity of the cornea, is called a *hernia of the cornea or keratocele.* In order to understand its origin and nature, we must briefly consider the anatomical changes which take place in the tissues of the cornea, in case of ulceration. Proceeding from the anterior surface of the cornea the ulceration destroys successively the anterior epithelium, Bowman's membrane, and the several layers of the corneal tissue proper. Now, the first indication of a pathological (inflammatory or ulcerative) process going on in these structures, is the loss of transparency; they become opaque, and therefore an ulcer which has not gone beyond the fibrillar tissue of the cornea, presents a gray, grayish white, or yellowish gray color. But below these layers there is the so-called membrane of Descemet. This is a structureless, very elastic membrane, lined with one layer of pavement epithelium, the posterior epithelium of the cornea. This membrane is not easily affected by ulceration, and, owing to its structureless nature, preserves its transparency; and when a corneal ulcer has extended in depth as far as the membrane of Descemet, its floor suddenly becomes transparent and clear, like an ulcer at the time when the destruction has ceased and all molecular débris has been removed, and the process of reparation has commenced. And, gentlemen, in this stage a corneal ulcer has often been a source of deception to both physician and patient. The inexperienced physician, noticing the remarkable change in the appearance of the ulcer, does not recognize the real, dangerous character of this change, and prognosticates a very favorable, speedy recovery, when to his terror a few hours or days later he finds the cornea perforated. And patients also are often misled. Suppose the ulceration occupies the center of the cornea; its opacity obscures the pupil and impairs the sight; the deeper the ulcer, the denser the opacity and the greater the impairment of vision. Now, picture to yourselves the happy surprise of a man who for weeks has been almost blind with a central ulcer of the cornea, at suddenly finding that he can see again pretty well with that eye. He, of course greets this change with joy as the sure sign of the long looked-for crisis toward recovery ; he laughs at the serious looks of his medical adviser who is far from sharing his patient's hopefulness, but on the contrary earnestly enjoins double caution, lest the eye might be suddenly destroyed. The patient is disposed to think that all danger is removed, since his sight has been restored, and further precaution is unnecessary. But suddenly something gives way, the patient feels a violent pain through his eye, a few drops of water run down his cheek, and the sight is gone, perhaps forever. Descemet's membrane which formed the transparent basis of the ulcer while the patient was enjoying the sudden return of his sight, had yielded to the pressure of the aqueous humor, it ruptured, and a perforation of the cornea was established. This is the usual result of deep progressive ulceration of the cornea.

But when the ulcer is very small the membrane of Descemet may, for some time, form the basis of the ulcer, and under favorable circumstances the cornea may escape the fate of perforation. In these cases a small portion of Descemet's membrane, (which, as I said before, is very elastic) is crowded into and through the tunnel-like canal of the ulcer by the pressure of the aqueous humor, just as the peritoneum is pushed into the inguinal canal in the case of hernia. Therefore the name "hernia of the cornea" is given to this affection, although strictly speaking it is a protrusion of Descemet's membrane alone. Such is the condition of this patient's right cornea. This transparent and prominent spot represents a small pouch of the membrana Descemeti, protruding through a deep, crater-like ulcer of the cornea. The patient, 13 years of age, has granular conjunctivitis, in the course of which ulceration of the cornea is a common complication. And one of these ulcers deepened until it reached the posterior glass membrane. You cannot see the depth of the ulcer because its space is filled by this hernial sac which comes even with, or rather rises a little above the anterior surface of the cornea. But if I should puncture this vesicle with the point of a needle you would see the whole aqueous humor escape, the sac collapse, and a deep defect in the cornea appear in its place.

Usually in cases of this kind the thin membrane of the keratocele is gradually stretched to its utmost capacity, and finally gives way to the continued pressure of the aqueous humor. This fluid then escapes, the iris falls against the perforation, and is permanently fastened there by agglutination. An adherent leucoma, with all its unpleasant, often disastrous consequences, is the ultimate result.

From these observations, it is obvious what our chief object in the treatment of this kesatocele must be. We must endeavor to reduce the hernia, to prevent, if possible, the occurrence of perforation, and to heal the ulcer of the cornea.

If we possessed a medicine which could so effectually diminish the pressure of the aqueous humor that the membrana Descemeti could resist it without bulging, this remedy would admirably fulfill the indications in our case. It would reduce the hernia of the cornea, and by its reposition would remove the greatest obstacle to a speedy repair of the ulcerated cornea. For as long as the hernial sac occupies the gap of the corneal ulceration, it prevents, like a wedge, its edges from approaching each other, and renders it utterly impossible that granulations can sprout up from the sides and repair the defect. The same remedy would also remove all danger of perforation, because the minute the pressure of the aqueous humor is diminished, all strain which could occasion a rupture, is taken from the membrana Descemeti. Until quite recently, such remedy was unknown to ophthalmic surgeons, and the treatment of a hernia of the cornea consisted in the application of pressure bandage, repeated paracentesis of the cornea, or iridectomy.

The *pressure bandage* should act like a truss; it should overcome the pressure of the aqueous humor upon the posterior surface of the cornea by a counter pressure upon its anterior surface; it should push the hernia back and prevent its coming out again.

*Paracentesis of the cornea* diminishes the pressure upon the posterior surface of the cornea by withdrawing the aqueous humor. If you take a tensely-filled bladder and let out a part of the contents, its walls will become flaccid with the decrease of tension. So, if you drain off the aqueous humor, the tissues of the eye in general, and the cornea in particular, are relieved of a certain amount of the pressure which the contents of the globe (aqueous humor, crystalline lens and vitreous body) exert upon them. But the relief afforded by paracentesis is of a very transient nature, because the anterior chamber is speedily refilled with aqueous humor, and, pari passu, the original pressure restored. Therefore the operation must be often repeated if the pressure is to be kept below the normal degree for any length of time.

*Iridectomy* is known to permanently lower the intra-ocular pressure, and for this reason most surgeons consider it preferable to paracentesis in all cases in which a prolonged diminution of pressure is indicated.

Within the past three years, however, ophthalmic surgeons have become acquainted with two remedies which seem likely to play an important rôle in the treatment of deep ulcerations of the cornea, and other affections which call for a reduction of the intra-ocular pressure. These two remedies are, the alkaloid of the calabar bean, called *eserine*, and the alkaloid of jaborandi, called *pilocarpine*. The sulphate of eserine and the muriate of pilocarpine are the preparations now used in ophthalmic practice. They are both quite soluble in water, making a clear, colorless solution; but in a warm temperature the solution of eserine turns red, and becomes inefficient, while the solution of pilocarpine does not change in color and efficiency. This solution (0.05 in 25 grams of water) before you, has been prepared two months ago; it is perfectly clear yet, and as effective as on the first day.

If you put a drop of it in your eye, you feel a faint smarting sensation, and notice a slight blush of the conjunctiva. But this irritation passes away quickly. After ten minutes the pupil is contracted, and distant objects appear indistinct; in twenty minutes the pupil is constricted to a small pin hole, and distant vision is exceedingly indistinct, but can be cleared up by strong concave glasses. In other words, the eye has become highly near-sighted. The myopia is induced by spasm of the ciliary muscle, and the contraction of the pupil (myosis) by spasm of the sphincter pupillæ. Both alkaloids seem to have the same effect upon the eye; only pilocarpine produces less conjunctival irritation than eserine.

For our present purpose it is particularly interesting to learn that simultaneously with the spasm of the intra-ocular muscles the intra-ocular tension is diminished. In 1876, Laqueur, of

Strassburg, reported * that he observed a diminution of the hardness of glaucomatous eyeballs after the instillation of eserine. In the same year, Adolph Weber, of Darmstadt, published † a very interesting paper, "On Calabar and its Therapeutical Utility."

With a very sensitive instrument (tonometer) he had carefully tested the degree of tension of the cornea before and after the application of extract of calabar bean. A long series of experiments, made in 1869, convinced him of the fact that he had found in the calabar bean a drug which decidedly lessened the pressure upon the posterior surface of the cornea. His conviction was further strengthened by an observation which fully confirmed his experimental studies. In a case of keratocele he observed that after application of calabar the vesicle of the protruding hernia sank back to the basis of the corneal ulcer; and under the continued use of calabar the protrusion of the membrana Descemeti did not return; the ulcer was filled with new tissue and covered with new and transparent epithelium; the eye recovered, and a ring-shaped cicatrix, with a transparent center, was the only trace the keratocele had left. Besides, in keratocele, Dr. Weber found the extract of calabar bean (which he used till 1875, when he substituted the alkaloids, eserine and pilocarpine) most useful in deep, progressive ulcers of the cornea, conical cornea, staphyloma, and peripheric prolapse of the iris.

Wecker, of Paris, who first introduced the alkaloids in ophthalmic practice, fully confirmed ‡ Weber's observations and therapeutical indications of the use of eserine and pilocarpine.

Henry W. Williams, § of Boston, has during the past two years made extensive use of eserine and pilocarpine in the treatment of corneal ulcers. His experience not only coincided with that of the other authors, but he also added superficial keratitis to the affections for which the alkaloids are indicated. "In phlyctenular or herpetic eruptions of the conjunctiva, or of the epithelial layer of the cornea, eserine is of service, especially when

---

* Centralbl. d. Medicin. Wissenschaften, 1876, No. 24.

† Graefe's Archiv. f. Ophthalmologie, Band XXII, 4.

‡ Klin. Monatsbl. f. Augenheilkunde, 1877, p. 60.

§ Boston Med. and Surgical Journal, March 14, '78.

photophobia is present, and is far preferable to atropia, which, by causing intolerance of light, adds to the patient's discomfort."

The unanimous testimony of these and other observers seems to point out clearly enough the course of treatment we should pursue in our case. And I will tell you that we tried this plan, but were obliged to abandon it soon again. The boy was admitted to the infirmary on the first day of August. The cornea was hazier and the keratocele a little larger than it is now, and the whole upper half of the cornea appeared to bulge out. Pilocarpine was dropped in the eye in the morning and evening. After two days the upper half of the cornea showed the same curvature as the lower half, and the hernia appeared somewhat sunken in. Under the continuance of pilocarpine, however, the eye became very painful, and red around the cornea, and a fresh ulcer made its appearance in the center of the cornea. These unfavorable symptoms increased in degree so much that on the fifth day it was thought best to discontinue the use of pilocarpine. We applied the pressure bandage, and the eye quickly recovered from the attack. In the middle of August a second trial with pilocarpine had the same result. The eye did not seem disposed to tolerate the new treatment; and we had to fall back upon the old resources of pressure bandage and iridectomy. Under the continued use of the bandage, the eye improved in so far as the lower half of the cornea cleared up sufficiently to afford us a pretty good view of the iris and pupil. We could then ascertain that the pupil was contracted and the edge of the iris firmly fastened to the anterior surface of the crystalline lens.

This state of the pupil fully accounted for the intolerance the eye had shown for the pilocarpine treatment. It is a well established experience that an eye with extensive or circular posterior synechiæ as the result of iritis, generally does not well bear any stimulating, topical treatment. And it is evident that pilocarpine, the most powerful stimulant for the musculature of the iris must have a bad effect on an eye which has just recovered from an inflammation of the iris.

The failure of pilocarpine in our case, therefore, only confirms the experience of others to the effect that the use of this remedy is contra-indicated in affections of the iris, and consequently also in corneal troubles when the iris is implicated.

The condition of the iris and pupil at once settled the question what the next step should be in the treatment of our case. For reasons which I shall discuss at some future occasion, circular synechiæ of the iris are regarded as a strong indication for the performance of iridectomy. And, as I have stated before, the same operation is chosen by most surgeons if an operative interference is deemed necessary in case of keratocele. The two conditions being united in our case, there could be no doubt but what iridectomy should be performed; and the only question to be decided was in regard to the locating of the artificial pupil. The greater transparency of the lower half of the cornea suggested a downward iridectomy, because the operation could be done under more favorable conditions, and would probably at once restore a pretty good sight. But then, a coloboma of the lower portion of the iris is a greater blemish to the eye than a defect in the upper half of the iris, because the latter is to a great extent concealed by the upper lid. Besides, the upper half of the cornea was not so cloudy that the point of an instrument in the anterior chamber could not be seen without difficulty; and it was very likely that after the operation this haziness would gradually disappear, and the upper part of the cornea would become as clear as the lower. And finally, if a piece of the upper half of the iris was excised, the iris was definitely removed from behind the keratocele, and could not be caught in a perforation of the cornea if the hernial sac should burst after all.

These reasons induced me to perform an upward iridectomy on September 19. The eye recovered quickly from the immediate effect of the operation, and has never since shown any signs of irritation, although occasionally astringent applications were made upon the thickened and congested conjunctiva. Under the continued use of the pressure bandage the protrusion of Descemet's membrane has decidedly diminished in degree and circumference. This sign, of course, is the best indication that the eye is improving and progressing favorably toward recovery.

